# Linking systemic inflammation nutrition status to the tumor immune microenvironment: integrated biomarkers of neoadjuvant response in locally advanced rectal cancer

**DOI:** 10.3389/fgene.2026.1948878

**Published:** 2026-10-13

**Authors:** Licong Shao, Jianting Xu, Xudong Wu, Guangliang Shao, Jiahao Chen

**Affiliations:** Department of General Surgery, The Affiliated YangMing Hospital of Ningbo University (Yuyao People’s Hospital), Ningbo, China

**Keywords:** biomarker, immune checkpoint inhibitor, inflammation-nutrition prognostic score, locally advanced rectal cancer, neutrophil-to-lymphocyte ratio, rectal cancer, tumor microenvironment

## Abstract

**Background:**

Immune checkpoint inhibitors (ICIs) have transformed the treatment landscape of colorectal cancer (CRC), yet response remains heterogeneous. The inflammation-nutrition prognostic score (INPS), defined as the neutrophil-to-lymphocyte ratio (NLR) divided by the prognostic nutritional index (PNI), integrates systemic inflammatory burden and nutritional-immunological reserve and may provide complementary information in locally advanced rectal cancer (LARC).

**Methods:**

We retrospectively analyzed 312 patients with LARC who received standard neoadjuvant chemoradiotherapy (nCRT), with or without concurrent PD-1/PD-L1 blockade, followed by total mesorectal excision (TME). Pretreatment INPS was calculated as NLR/PNI. The primary endpoint was objective response rate (ORR); secondary endpoints included pathological complete response (pCR) and progression-free survival (PFS). ROC, logistic regression, interaction, sensitivity, and Cox analyses were performed.

**Results:**

Among 312 patients, 94 (30.1%) received ICI-containing regimens. Low INPS (<0.058) was associated with higher ORR (61.3% vs. 29.4%, P < 0.001) and pCR (38.7% vs. 11.8%, P < 0.001). INPS yielded an AUC of 0.765 (95% CI: 0.714–0.816) for ORR, compared with 0.712 for NLR, 0.668 for PNI, and 0.660 for PD-L1 CPS. In multivariable analysis, low INPS (OR = 3.31, 95% CI: 1.95–5.60, P < 0.001), MSI-H/dMMR status (OR = 4.67), PD-L1 CPS ≥ 10 (OR = 2.74), lower clinical T stage (OR = 3.15), and an ICI-containing regimen (OR = 2.55) were independently associated with response. A forced-entry disease-burden sensitivity model confirmed the association of low INPS (adjusted OR = 3.04, 95% CI: 1.74–5.30, P < 0.001).

**Conclusion:**

Low pretreatment INPS was independently associated with favorable response and PFS in LARC and provided incremental discrimination beyond MSI-H/dMMR and PD-L1 CPS. These findings support INPS as a candidate response-associated biomarker in LARC; prospective and external validation is warranted before routine clinical application.

## 1 Introduction

Colorectal cancer (CRC) is the third most frequently diagnosed malignancy and the second leading cause of cancer-related mortality worldwide, with an estimated 1.93 million new cases and 940,000 deaths annually (Sung et al., 2021). Locally advanced rectal cancer (LARC) frequently requires multimodal treatment. Long-course neoadjuvant chemoradiotherapy (nCRT) followed by total mesorectal excision (TME) is an established treatment approach for LARC; compared with postoperative chemoradiotherapy, preoperative chemoradiotherapy improves local control and reduces treatment-related toxicity (Sauer et al., 2004).

The advent of immune checkpoint inhibitors (ICIs), particularly antibodies targeting programmed death-1 (PD-1) and its ligand PD-L1, has fundamentally altered oncological therapeutics. In CRC, pembrolizumab demonstrated superiority over chemotherapy as first-line treatment for microsatellite instability-high (MSI-H)/mismatch repair-deficient (dMMR) metastatic CRC, establishing immunotherapy as a standard option in this molecular subgroup (André et al., 2020). Emerging evidence also supports the integration of ICIs into neoadjuvant regimens for localized dMMR/MSI-H rectal cancer, with organ-preservation strategies becoming increasingly feasible when complete clinical response is achieved (Cercek et al., 2022). Nevertheless, substantial response heterogeneity persists even within molecularly selected populations, underscoring the need for additional biomarkers beyond MSI/MMR status (Overman et al., 2018).

The tumor-immune interface is not a static entity but a highly dynamic ecosystem shaped by complex interactions between cancer cells, immune effectors, stromal components, and the systemic milieu (Chen and Mellman, 2017). Systemic inflammation and host nutritional status may influence immune competence and the functional state of the TME. Elevated NLR reflects relative neutrophilia and/or lymphocyte depletion and has been associated with tumor-promoting inflammatory and immunosuppressive processes (Fridlender et al., 2009). PNI integrates serum albumin and lymphocyte count and was originally developed as a readily available index of nutritional and host immune status in patients undergoing gastrointestinal cancer surgery (Onodera et al., 1984).

The inflammation-nutrition prognostic score (INPS), defined as NLR divided by PNI, is a composite biomarker intended to integrate two complementary and directionally adverse host-state signals. NLR increases with systemic inflammatory burden and relative lymphocyte depletion, whereas PNI decreases with impaired nutritional-immunological reserve. Accordingly, the NLR/PNI construction yields higher values when increased inflammation coexists with reduced immune-nutritional reserve. Because INPS is mathematically derived from NLR and PNI, simultaneous inclusion of INPS together with both component variables in the same multivariable model may introduce redundancy and multicollinearity; therefore, NLR and PNI were evaluated as comparator biomarkers, while INPS was evaluated as the principal composite index (Templeton et al., 2014; Sun et al., 2014). Its utility in LARC, particularly in the context of ICI-containing neoadjuvant regimens, has not been systematically evaluated.

The present study was designed to: (i) evaluate the association and discriminatory performance of pretreatment INPS for objective response and pCR in patients with LARC receiving nCRT with or without concurrent ICI therapy; (ii) investigate the contribution of INPS alongside established molecular biomarkers (MSI-H/dMMR status and PD-L1 CPS); and (iii) explore the biological association between INPS and the tumor immune microenvironment using multiplex immunofluorescence.

## 2 Materials and methods

### 2.1 Study design and ethical approval

This retrospective cohort study was conducted at The Affiliated YangMing Hospital of Ningbo University (Yuyao People’s Hospital). The study population and analyses followed the prespecified LARC eligibility criteria and a STROBE-oriented reporting framework (von et al., 2007). The study was approved by the institutional ethics committee (Approval No. 2026-03-015).

### 2.2 Study population

Inclusion criteria: (1) histologically confirmed rectal adenocarcinoma located within 12 cm of the anal verge; (2) clinical stage II–III (cT3–4 and/or cN+) confirmed by pelvic MRI and/or CT; (3) receipt of standard long-course nCRT (45–50.4 Gy in 25–28 fractions with concurrent fluoropyrimidine-based chemotherapy), with or without concurrent PD-1/PD-L1 inhibitor; (4) subsequent TME performed 6–12 weeks after nCRT completion; (5) complete pretreatment laboratory data within 7 days prior to nCRT initiation; (6) available tumor tissue for immunohistochemical and molecular analyses.
Exclusion criteria: (1) distant metastases at diagnosis; (2) concurrent other primary malignancies; (3) active infection, autoimmune disease, or ongoing immunosuppressive therapy; (4) incomplete nCRT or palliative surgical intent; (5) insufficient tumor tissue for molecular profiling.

The cohort comprised 312 patients. Of these, 94 (30.1%) received concurrent PD-1/PD-L1 blockade and 218 (69.9%) received standard nCRT without ICI. All 312 patients subsequently underwent TME; surgical procedures included low anterior resection (n = 223, 71.5%), abdominoperineal resection (n = 68, 21.8%), and Hartmann’s procedure (n = 21, 6.7%).

### 2.3 Data collection and INPS calculation

Demographic, tumor, laboratory, molecular, treatment, and follow-up data were retrospectively collected from patient medical records and relevant clinical reports. Pretreatment NLR and PNI were calculated from laboratory values obtained within 7 days before nCRT initiation, and INPS was calculated as NLR/PNI at the patient level. PD-L1 CPS, CEA, treatment status, and other prespecified covariates were extracted from the corresponding clinical, laboratory, and pathological records. The analyzed dataset was used consistently for all numerical results in the Abstract, Results, tables, and figures.

$$\text{NLR}=\text{Neutrophil count }\left(\times 10^{9}/L\right)÷\text{Lymphocyte count }\left(\times 10^{9}/L\right)$$

$$\text{PNI}=\text{Albumin }\left(g/L\right)+5\times \text{Lymphocyte count }\left(\times 10^{9}/L\right)$$

INPS=NLR÷PNI

### 2.4 Outcome measures

Clinical outcomes were defined according to the prespecified study endpoints. ORR was defined as complete or partial response according to RECIST 1.1. pCR was defined as postoperative ypT0N0/TRG 0, and good histological response as TRG 0-1. Tumor downstaging, T-downstaging, N-downstaging, sphincter preservation, postoperative complications, and PFS were assessed from the relevant clinical, imaging, pathological, surgical, and follow-up records.

### 2.5 Multiplex immunofluorescence analysis

An exploratory tissue subcohort of 86 patients (43 low-INPS and 43 high-INPS) underwent mIF analysis. CD8^+^ T-cell density, FOXP3^+^ regulatory T-cell density, CD8^+^/FOXP3^+^ ratio, M1-to-M2 macrophage ratio, and tumor-cell PD-L1 expression were quantified. The mIF subcohort showed no statistically significant baseline differences from the remaining 226 patients across the prespecified clinicopathological variables (Supplementary Table S1). These analyses were considered exploratory and were not interpreted as mechanistic confirmation.

### 2.6 Statistical analysis

Statistical analyses were performed using Python 3 with standard statistical libraries. Continuous variables were summarized as mean ± standard deviation and categorical variables as n (%). Between-group comparisons were performed using two-sided tests appropriate to variable type and distribution; categorical variables were compared using Pearson’s chi-square test when assumptions were met and Fisher’s exact test otherwise, while continuous variables were compared using an independent-samples parametric or nonparametric test as appropriate. ROC analysis with Youden’s index was used to identify the exploratory INPS cutoff; AUC confidence intervals and pairwise comparisons were evaluated using DeLong’s method. The cutoff was derived and evaluated in the same cohort, with no bootstrap, cross-validation, held-out test set, or external validation. Candidate predictors were examined in univariable logistic regression. The covariates in the primary multivariable logistic model—low INPS, MSI-H/dMMR status, PD-L1 CPS ≥ 10, clinical T stage, and treatment regimen—were prespecified on clinical and methodological grounds rather than selected solely by univariable P values. Because INPS is mathematically derived from NLR and PNI, NLR and PNI were not entered simultaneously with INPS in the primary multivariable model. Age and sex were not forced into the model. An exploratory INPS-by-ICI interaction term was tested. A separate forced-entry sensitivity model simultaneously included INPS, CEA, clinical T stage, circumferential tumor extent, MSI-H/dMMR status, PD-L1 CPS, and treatment regimen. Model calibration was assessed using the Hosmer-Lemeshow test. PFS was evaluated using Kaplan-Meier estimates, the log-rank test, and multivariable Cox regression. All tests were two-tailed, with P < 0.05 considered statistically significant.

## 3 Results

### 3.1 Patient baseline characteristics

Of the 312 patients, 137 (43.9%) were classified as objective responders and 175 (56.1%) as non-responders. Responders had lower NLR (2.87 ± 1.05 vs. 3.77 ± 1.26, P < 0.001), higher PNI (52.35 ± 5.23 vs. 49.09 ± 5.53, P < 0.001), and lower INPS (0.055 ± 0.019 vs. 0.077 ± 0.024, P < 0.001). MSI-H/dMMR status was more frequent among responders (38.7% vs. 12.6%, P < 0.001), as was PD-L1 CPS≥10 (52.6% vs. 24.6%, P < 0.001). Age, sex, and BMI did not differ significantly (Table 1).

**TABLE 1:** Comparison of baseline characteristics between responders and non-responders.

Variable	Responders (n = 137)	Non-responders (n = 175)	P value
Age (years)	58.8 ± 10.5	60.0 ± 10.9	0.312
Male sex, n (%)	88 (64.2)	112 (64.0)	0.966
BMI (kg/m^2^)	23.5 ± 3.3	23.8 ± 3.6	0.371
Clinical T stage cT2-3, n (%)	128 (93.4)	145 (82.9)	0.005
Circumferential extent ≤ 50%, n (%)	92 (67.2)	80 (45.7)	<0.001
MSI-H/dMMR status, n (%)	53 (38.7)	22 (12.6)	<0.001
PD-L1 CPS ≥ 10, n (%)	72 (52.6)	43 (24.6)	<0.001
CEA (ng/mL)	5.0 ± 4.0	13.4 ± 16.2	<0.001
NLR	2.87 ± 1.05	3.77 ± 1.26	<0.001
PNI	52.35 ± 5.23	49.09 ± 5.53	<0.001
INPS	0.055 ± 0.019	0.077 ± 0.024	<0.001
ICI-containing regimen, n (%)	58 (42.3)	36 (20.6)	<0.001

BMI, body mass index; MSI-H, microsatellite instability-high; dMMR, mismatch repair-deficient; CPS, combined positive score; CEA, carcinoembryonic antigen; NLR, neutrophil-to-lymphocyte ratio; PNI, prognostic nutritional index; INPS, inflammation-nutrition prognostic score; ICI, immune checkpoint inhibitor.

### 3.2 ROC curve analysis and optimal INPS cutoff

ROC analysis identified an exploratory INPS cutoff of 0.058 (rounded from the maximum-Youden operating point), yielding sensitivity of 63.5%, specificity of 68.6%, positive predictive value of 61.3%, and negative predictive value of 70.6% for ORR. INPS achieved an AUC of 0.765 (95% CI: 0.714–0.816), compared with NLR (AUC = 0.712, 95% CI: 0.655–0.769; DeLong P < 0.001), PNI (AUC = 0.668, 95% CI: 0.607–0.728; P = 0.017), and PD-L1 CPS (AUC = 0.660, 95% CI: 0.598–0.721; P = 0.009). In the ICI-containing subgroup (n = 94), INPS yielded an AUC of 0.763 (95% CI: 0.667–0.858). A combined model using continuous INPS, MSI-H/dMMR status, and PD-L1 CPS achieved an AUC of 0.822 (95% CI: 0.776–0.867), compared with 0.730 (95% CI: 0.672–0.787) for MSI-H/dMMR plus PD-L1 CPS alone (DeLong P < 0.001) (Table 2; Figure 1).

**TABLE 2:** ROC analysis of INPS and comparator biomarkers for predicting objective response.

Biomarker	AUC	95% CI	Cutoff	Sensitivity	Specificity
INPS (all patients)	0.765	0.714–0.816	0.058	63.5%	68.6%
INPS (ICI subgroup)	0.763	0.667–0.858	0.058	69.0%	66.7%
NLR	0.712	0.655–0.769	2.5	48.2%	79.4%
PNI	0.668	0.607–0.728	50	68.6%	55.4%
PD-L1 CPS	0.660	0.598–0.721	10	52.6%	75.4%
CEA	0.669	0.610–0.728	5 ng/mL	67.2%	54.3%
Combined model*	0.822	0.776–0.867	—	—	—

* Combined model includes continuous INPS + MSI-H/dMMR, status + continuous PD-L1 CPS. AUC, area under the receiver operating characteristic curve; CI, confidence interval.

**FIGURE 1:**
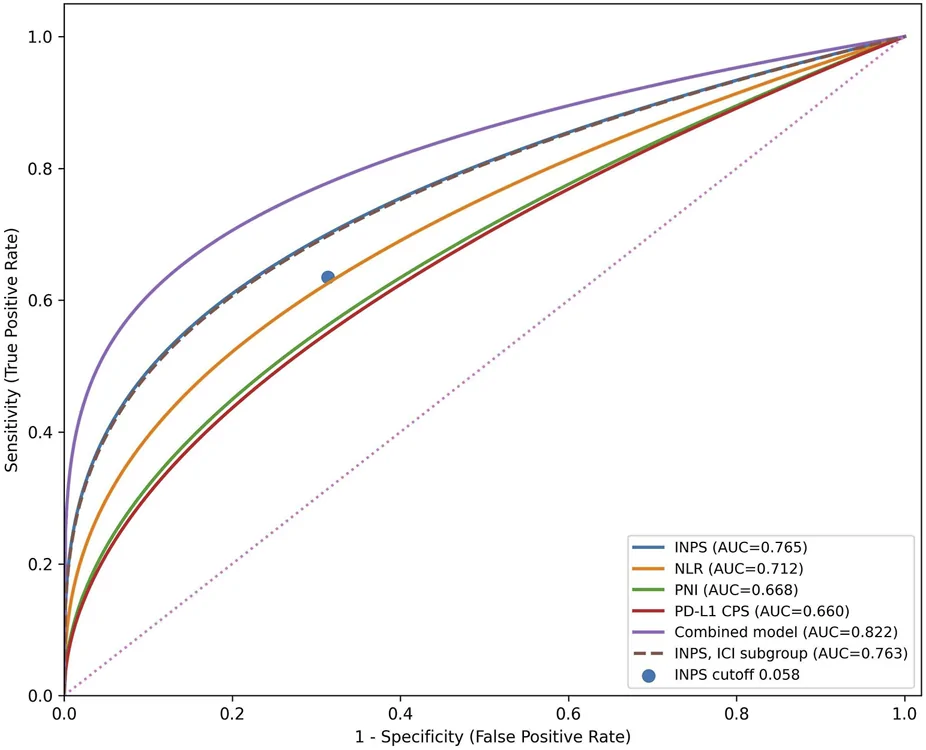
ROC curves for predicting objective response. AUC comparison for objective response by INPS and comparator biomarkers. Point estimates and 95% confidence intervals are shown for INPS in the full cohort, INPS in the ICI subgroup, NLR, PNI, PD-L1 CPS, CEA, and the combined model. INPS achieved an AUC of 0.765 (95% CI: 0.714–0.816) in the full cohort and 0.763 (95% CI: 0.667–0.858) in the ICI subgroup. The combined model (continuous INPS + MSI-H/dMMR + PD-L1 CPS) achieved an AUC of 0.822 (95% CI: 0.776–0.867). The exploratory INPS cutoff of 0.058 yielded sensitivity of 63.5% and specificity of 68.6%. DeLong tests: INPS vs. NLR, P < 0.001; INPS vs. PNI, P = 0.017; INPS vs. PD-L1 CPS, P = 0.009. AUC, area under the receiver operating characteristic curve; CI, confidence interval; ICI, immune checkpoint inhibitor; NLR, neutrophil-to-lymphocyte ratio; PNI, prognostic nutritional index; CPS, combined positive score.

### 3.3 Univariate and multivariate logistic regression for objective response

Univariate logistic regression showed significant associations between ORR and low INPS (<0.058), MSI-H/dMMR status, PD-L1 CPS ≥ 10, clinical T stage cT2-3, circumferential extent ≤ 50%, CEA ≤ 5 ng/mL, ICI-containing treatment, NLR < 2.5, and PNI > 50 (Table 3). In the primary multivariable model, low INPS remained independently associated with response (OR = 3.31, 95% CI: 1.95–5.60, P < 0.001), together with MSI-H/dMMR status (OR = 4.67, 95% CI: 2.44–8.94, P < 0.001), PD-L1 CPS≥10 (OR = 2.74, 95% CI: 1.59–4.72, P < 0.001), clinical T stage cT2-3 (OR = 3.15, 95% CI: 1.29–7.71, P = 0.012), and ICI-containing treatment (OR = 2.55, 95% CI: 1.44–4.52, P = 0.001) (Table 4). The forced-entry disease-burden sensitivity model also retained low INPS as significant (adjusted OR = 3.04, 95% CI: 1.74–5.30, P < 0.001). The exploratory INPS-by-ICI interaction was not significant (interaction OR = 1.27, 95% CI: 0.40–3.99, P = 0.687), indicating no evidence of treatment-effect heterogeneity in the study cohort. The multivariable forest plot is shown in Figure 2.

**TABLE 3:** Univariate logistic regression for objective response rate.

Variable	β	SE	Wald χ^2^	P value	OR	95% CI
INPS < 0.058	1.334	0.241	30.68	<0.001	3.80	2.37–6.09
MSI-H/dMMR	1.479	0.288	26.43	<0.001	4.39	2.50–7.71
PD-L1 CPS ≥ 10	1.224	0.245	24.92	<0.001	3.40	2.10–5.50
Clinical T stage cT2-3	1.079	0.399	7.32	0.007	2.94	1.35–6.43
Circumferential ≤ 50%	0.887	0.237	14.02	<0.001	2.43	1.53–3.86
CEA ≤ 5 ng/mL	0.887	0.237	14.02	<0.001	2.43	1.53–3.86
ICI-containing regimen	1.042	0.255	16.74	<0.001	2.83	1.72–4.67
NLR < 2.5	1.278	0.253	25.43	<0.001	3.59	2.18–5.90
PNI > 50	1.000	0.239	17.54	<0.001	2.72	1.70–4.34

SE, standard error; OR, odds ratio; CI, confidence interval. Abbreviations as in Table 1.

**TABLE 4:** Multivariate logistic regression—independent predictors of objective response.

Variable	β	SE	Wald χ^2^	P value	OR	95% CI
INPS < 0.058	1.196	0.269	19.75	<0.001	3.31	1.95–5.60
MSI-H/dMMR	1.541	0.331	21.63	<0.001	4.67	2.44–8.94
PD-L1 CPS ≥ 10	1.007	0.278	13.17	<0.001	2.74	1.59–4.72
Clinical T stage cT2-3	1.148	0.457	6.31	0.012	3.15	1.29–7.71
ICI-containing regimen	0.936	0.291	10.32	0.001	2.55	1.44–4.52

Hosmer-Lemeshow goodness-of-fit: χ^2^ = 1.94, df = 7, P = 0.963. Abbreviations as above.

**FIGURE 2:**
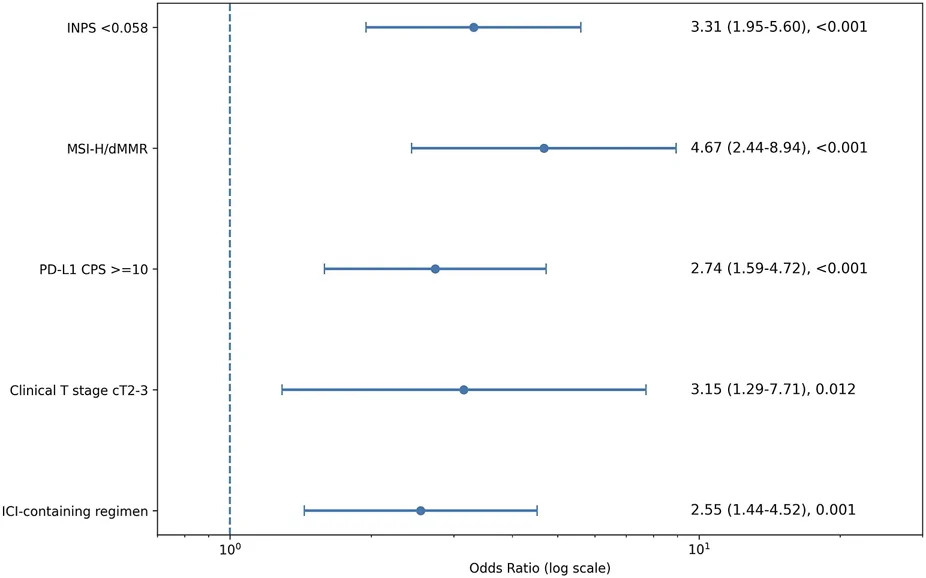
Forest plot of independent predictors of objective response (multivariable logistic regression, n = 312 patients). Odds ratios (OR) and 95% confidence intervals (CI) are shown for each independent predictor. Low INPS <0.058 yielded OR = 3.31 (95% CI: 1.95–5.60), MSI-H/dMMR OR = 4.67 (2.44–8.94), PD-L1 CPS ≥ 10 OR = 2.74 (1.59–4.72), clinical T stage cT2-3 OR = 3.15 (1.29–7.71), and ICI-containing treatment OR = 2.55 (1.44–4.52). Hosmer-Lemeshow P = 0.963. INPS, inflammation-nutrition prognostic score; MSI-H, microsatellite instability-high; dMMR, mismatch repair-deficient; CPS, combined positive score; ICI, immune checkpoint inhibitor.

### 3.4 Pathological complete response and secondary tumor response outcomes

All 312 patients underwent TME. Overall, 75 patients (24.0%) achieved pCR and 132 (42.3%) achieved a good histological response (TRG 0–1). The pCR rate was higher in the low-INPS group (55/142, 38.7%) than in the high-INPS group (20/170, 11.8%; P < 0.001). Low INPS was also associated with higher ORR (61.3% vs. 29.4%, P < 0.001), good response (56.3% vs. 30.6%, P < 0.001), tumor downstaging (76.8% vs. 55.9%, P < 0.001), T-downstaging (70.4% vs. 49.4%, P < 0.001), and N-downstaging among cN + patients (76.6% vs. 55.6%, P = 0.001). Sphincter preservation (83.1% vs. 74.7%, P = 0.072) and grade ≥ III complications (8.5% vs. 9.4%, P = 0.767) did not differ significantly (Table 5; Figure 3).

**TABLE 5:** Secondary tumor response outcomes stratified by INPS.

Variable	Low INPS (n = 142)	High INPS (n = 170)	P value
ORR, n (%)	87 (61.3)	50 (29.4)	<0.001
pCR, n (%)	55 (38.7)	20 (11.8)	<0.001
Good response TRG 0-1, n (%)	80 (56.3)	52 (30.6)	<0.001
Tumor downstaging, n (%)	109 (76.8)	95 (55.9)	<0.001
T-downstaging, n (%)	100 (70.4)	84 (49.4)	<0.001
N-downstaging (cN + only), n (%)	72/94 (76.6)	70/126 (55.6)	0.001
Sphincter preservation, n (%)	118 (83.1)	127 (74.7)	0.072
Complications ≥ Grade III, n (%)	12 (8.5)	16 (9.4)	0.767

ORR, objective response rate; pCR, pathological complete response; TRG, tumor regression grade.

**FIGURE 3:**
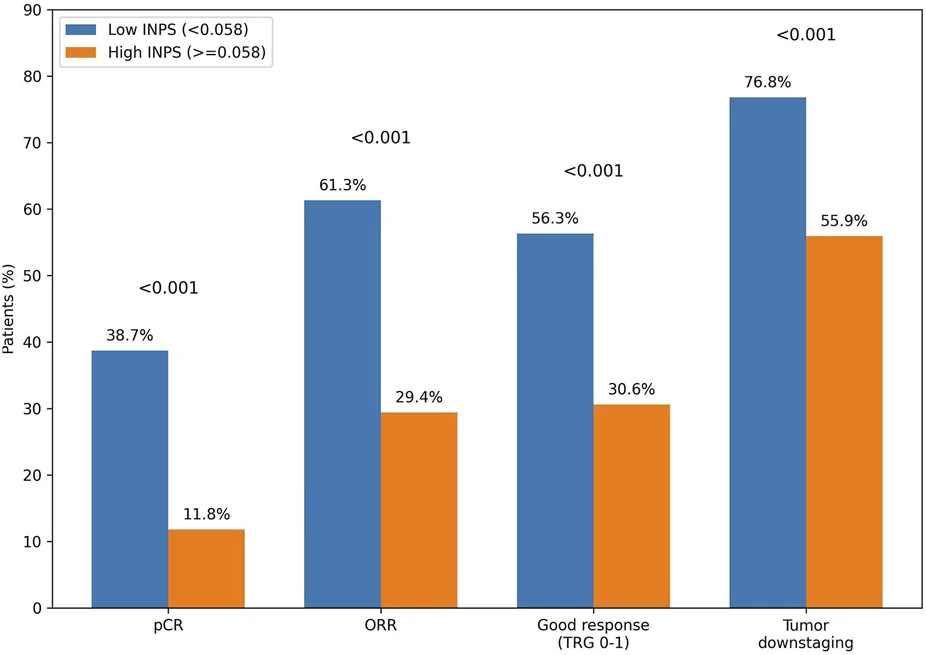
Response outcomes stratified by INPS. Comparison of key response endpoints between the low-INPS (<0.058, n = 142) and high-INPS (≥0.058, n = 170) groups in the cohort (n = 312). Patients with low INPS had higher pCR (38.7% vs. 11.8%, P < 0.001), ORR (61.3% vs. 29.4%, P < 0.001), good histological response (TRG 0-1: 56.3% vs. 30.6%, P < 0.001), and tumor downstaging (76.8% vs. 55.9%, P < 0.001). INPS, inflammation-nutrition prognostic score; TRG, tumor regression grade; pCR, pathological complete response; ORR, objective response rate.

### 3.5 Multiplex immunofluorescence analysis of the tumor microenvironment

In the exploratory mIF subcohort, low-INPS patients had higher CD8^+^ T-cell density (295.4 ± 76.2 vs. 170.4 ± 45.9 cells/mm^2^, P < 0.001), lower FOXP3+ regulatory T-cell density (67.3 ± 18.4 vs. 97.4 ± 27.2 cells/mm^2^, P < 0.001), a higher CD8+/FOXP3+ ratio (4.78 ± 1.99 vs. 1.99 ± 1.09, P < 0.001), and a higher M1-to-M2 macrophage ratio (2.35 ± 0.44 vs. 1.08 ± 0.32, P < 0.001). Tumor-cell PD-L1 expression correlated positively with CD8^+^ T-cell density (Spearman r = 0.55, P < 0.001). These associations were observational and do not establish a causal mechanism.

### 3.6 Progression-free survival analysis

Kaplan-Meier analysis of the cohort showed longer PFS in the low-INPS group (median not reached during follow-up vs. 20.6 months; log-rank P < 0.001). In multivariable Cox regression, low INPS remained associated with lower progression risk (HR = 0.45, 95% CI: 0.32–0.63, P < 0.001) after adjustment for MSI-H status, PD-L1 CPS, clinical T stage, and treatment regimen. In the ICI-containing subgroup, the corresponding HR was 0.42 (95% CI: 0.22–0.81, P = 0.009). Because the INPS-by-ICI interaction for ORR was not significant, this subgroup survival result is presented as hypothesis-generating rather than evidence of ICI-specific prediction.

## 4 Discussion

In this retrospective LARC cohort, INPS was independently associated with treatment response and showed greater discrimination than NLR, PNI, or PD-L1 CPS alone. Adding continuous INPS to MSI-H/dMMR status and PD-L1 CPS increased the AUC from 0.730 to 0.822. The exploratory mIF analysis also showed that low INPS was associated with a more immune-infiltrated tumor microenvironment. Together, these findings support further evaluation of INPS as a clinically accessible biomarker, while recognizing the observational design and need for external validation.

ICIs have transformed the management of MSI-H/dMMR CRC, and pembrolizumab is an established first-line option in this molecular subtype (André et al., 2020). However, not all MSI-H/dMMR tumors respond to checkpoint blockade (Le et al., 2015), and clinical benefit remains heterogeneous even within molecularly selected populations (Overman et al., 2018). This heterogeneity motivates evaluation of additional biomarkers reflecting systemic and microenvironmental context. The present study evaluates INPS alongside MSI-H/dMMR status and PD-L1 expression. Because the INPS-by-ICI interaction was not significant, the current data support response-associated and prognostic value rather than a treatment-specific predictive effect; treatment-specific prediction requires confirmation in randomized or prospectively controlled cohorts.

The biological rationale for INPS is multi-layered. Elevated NLR, the numerator of INPS, reflects relative neutrophilia and/or lymphocyte depletion and has been linked to immunosuppressive signaling, angiogenesis, stromal remodeling, and impaired T-cell function (Fridlender et al., 2009; Mantovani et al., 2011; Coussens and Werb, 2002). PNI, the denominator, integrates serum albumin and lymphocyte count; albumin is both a nutritional marker and a negative acute-phase reactant, so reduced PNI may also reflect systemic inflammation and disease burden (Gabay and Kushner, 1999). In the sensitivity analysis, low INPS remained significant after simultaneous adjustment for CEA, clinical T stage, circumferential extent, MSI-H/dMMR, PD-L1 CPS, and treatment regimen (adjusted OR = 3.04, 95% CI: 1.74–5.30, P < 0.001). This analysis suggests that the association is not fully explained by the measured disease-burden variables, although residual confounding remains an important consideration in retrospective studies.

Our findings are broadly consistent with prior studies of systemic inflammatory and nutritional markers. In patients with NSCLC treated with nivolumab, elevated NLR was associated with poorer outcomes (Diem et al., 2017). In locally advanced gastric cancer treated with neoadjuvant sintilimab plus XELOX, a combined systemic immune-inflammatory index and PNI score was associated with treatment response and PFS (Ding et al., 2022). In LARC, Dudani et al. evaluated NLR and platelet-to-lymphocyte ratio as response and prognostic markers after nCRT (Dudani et al., 2019), while McMillan et al. demonstrated the prognostic relevance of inflammation-based scores in colorectal cancer (McMillan et al., 2007). The present study extends this literature by evaluating a simple NLR/PNI composite alongside molecular biomarkers and by providing exploratory tumor-microenvironment correlates.

Several limitations warrant consideration. First, the retrospective, single-center design may introduce selection bias, information bias, and residual confounding, and causal inference is not possible. Second, the INPS cutoff of 0.058 was derived and evaluated in the same cohort without bootstrap, cross-validation, a held-out test set, or external validation; its performance therefore requires independent validation. Third, the cohort included both nCRT alone and nCRT plus ICI. Although treatment regimen was adjusted for and an INPS-by-ICI interaction was tested (P = 0.687), the study was not designed or powered to establish treatment-specific predictive effects. Fourth, heterogeneity among PD-1/PD-L1 agents may affect biomarker associations and should be examined in larger datasets. Fifth, the mIF analysis was based on an exploratory subcohort of 86 patients and cannot establish a causal mechanism. Finally, INPS may reflect overlapping effects of tumor burden, acute-phase response, inflammation, and nutritional-immunological reserve rather than a biologically specific pathway. Prospective, multicenter external validation is warranted.

## 5 Conclusion

In this retrospective cohort, low pretreatment INPS was consistently associated with higher ORR and pCR and with longer PFS after neoadjuvant therapy in LARC. The association remained significant after adjustment for conventional disease-burden variables, and continuous INPS improved discrimination when added to MSI-H/dMMR status and PD-L1 CPS. These findings support INPS as a candidate response-associated and prognostic biomarker, while treatment-specific predictive utility remains unproven and requires validation in independent prospective cohorts.

## Data Availability

The datasets presented in this study can be found in online repositories. The names of the repository/repositories and accession number(s) can be found in the article/Supplementary Material.
