# Supplementary material for: Linking systemic inflammation nutrition status to the tumor immune microenvironment: integrated biomarkers of neoadjuvant response in locally advanced rectal cancer
Source: Front Genet. 2026 Oct 13;17:1948878. doi: 10.3389/fgene.2026.1948878 (PMC13706675; doi:10.3389/fgene.2026.1948878)
Supplement: Supplementary file 1 [file Table1.docx]

**Supplementary Table S1**
*Baseline characteristics of the mIF subcohort versus patients not included in mIF analysis*

| Variable | mIF subcohort (n=86) | Non-mIF patients (n=226) | P Value |
| --- | --- | --- | --- |
| Age (years) | 58.92±10.26 | 59.72±10.88 | 0.548 |
| Male sex, n (%) | 57 (66.3) | 143 (63.3) | 0.621 |
| BMI (kg/m²) | 23.83±3.57 | 23.59±3.45 | 0.601 |
| Clinical T stage cT2-3, n (%) | 72 (83.7) | 201 (88.9) | 0.213 |
| Circumferential extent <=50%, n (%) | 43 (50.0) | 129 (57.1) | 0.261 |
| MSI-H/dMMR, n (%) | 22 (25.6) | 53 (23.5) | 0.694 |
| PD-L1 CPS >=10, n (%) | 31 (36.0) | 84 (37.2) | 0.854 |
| CEA (ng/mL) | 10.73±15.39 | 9.36±12.13 | 0.458 |
| NLR | 3.38±1.24 | 3.37±1.26 | 0.958 |
| PNI | 50.29±6.10 | 50.61±5.45 | 0.675 |
| INPS | 0.07±0.03 | 0.07±0.02 | 0.662 |
| ICI-containing regimen, n (%) | 30 (34.9) | 64 (28.3) | 0.259 |
| Objective response, n (%) | 33 (38.4) | 104 (46.0) | 0.224 |

*All comparisons are descriptive within the revised manuscript. No baseline variable differed significantly between the mIF and non-mIF groups (all P>0.05).*
